# What do Brazilian health professionals know about the frailty syndrome? A cross-sectional study

**DOI:** 10.1186/s12877-022-02927-6

**Published:** 2022-03-21

**Authors:** Wesley dos Reis Mesquita, Natalia Aquaroni Ricci

**Affiliations:** Master’s and Doctoral Programs in Physical Therapy, Universidade Cidade de São Paulo - UNICID, Rua Cesáreo Galeno, 448 Tatuapé, 03071–000 São Paulo, SP Brazil

**Keywords:** Aged, Frailty, Health professionals, Knowledge, Primary care

## Abstract

**Background:**

The growing care demand for frail older adults and those at risk of frailty in primary health care (PHC) requires professionals trained in the subject to promote adequate care. This study aimed to analyze the self-reported, theoretical and practical knowledge of PHC professionals about the frailty syndrome.

**Methods:**

This is an observational cross-sectional study with a sample of 485 Brazilian health professionals (bachelor’s degree) working in PHC with older adults. An electronic questionnaire was used to collect data on professional characteristics and self-reported, theoretical and practical knowledge concerning frailty phenotype. Agreement analysis between types of knowledge and multivariate logistic regression were performed to show the factors associated with knowledge about frailty.

**Results:**

Theoretical knowledge showed the worse result, with 87.5% of the professionals describing the syndrome incorrectly. Roughly half the professionals self-reported (52.6%) very little/no knowledge concerning the syndrome and demonstrated low practical knowledge (55.1%) when identifying clinical cases. There were misconceptions about the syndrome, like it is natural from the aging process (83.3%) and is synonymous with disability and comorbidity (51.2%). The majority of the professionals were unaware of instruments for assessing frailty (77.9%) and the phenotype criteria (68.2%). No agreement was observed between the types of knowledge. Professionals specialized in or who had taken training courses in older adult health were 6.1 and 2.7-fold more likely, respectively, to self-reported some knowledge on the frailty syndrome.

**Conclusions:**

PHC professionals presented little knowledge on the frailty syndrome. Most professionals were unaware of the frailty definition, its assessments for diagnosis and evidence for its treatment. The lack of knowledge on frailty could affect the care provided to older adults in primary care.

**Supplementary Information:**

The online version contains supplementary material available at 10.1186/s12877-022-02927-6.

## Background

Geriatric syndromes are multifactorial conditions, with high morbidity and associated with negative health outcomes [1]. Among syndromes, frailty deserves to be highlighted in the actions of primary health care (PHC) [2] since it is an emerging health problem [3], negatively impacts the lives of older adults [4], increases the demand for care, and generates high social and economic costs [5]. Data on 75,133 community-dwelling older adults in low- and middle-income countries showed the prevalence of 49% of pre-frailty and 17% of frailty [6].

PHC is the patients first contact within the health care system and ideally, the best service to provide care for the older adult population [7]. However, it is known that the majority of PHC professionals have general training and are not specialists in older adult care. Even among specialized professionals, there are disagreements regarding frailty definition [8, 9].

The frailty syndrome can be identified, managed, prevented and even reversed [10]. But the lack of knowledge on this topic can significantly affect the care provided to older adults. Thus, professionals who provide care to frail older adults or those at risk of developing the syndrome need specific skills and information on this condition, so that they can implement effective actions in the PHC [11].

Despite the large amount of research available on the frail population, little is known about how health professionals work with frailty in clinical practice [12]. Considering the growing demand in PHC for older adult care and the impact of the frailty on this population, the first step is to identify the knowledge that professionals have on this subject, to promote future actions to improve clinical practice and reduce the negative impact of the syndrome. Thus, this study aimed to analyze the self-reported, theoretical and practical knowledge of PHC professionals concerning the frailty syndrome.

## Methods

An observational cross-sectional study was conducted on PHC in the state of Minas Gerais (MG), Brazil. MG has the largest PHC coverage in the Brazilian public health care system (SUS), covering 79.4% of the state’s population [13]. This research was approved by the institutional research ethics committee.

### Sample

The inclusion criteria were any professional with a bachelor’s degree working in PHC for at least 6 months and caring for older adults (≥ 60 years) in clinical practice. Professionals absent from their practical activities (e.g. management, sick leave, vacation) or who refused to participate were excluded. Only professionals who met the eligibility criteria and electronically signed the consent form were directed to the questionnaire.

The sample calculation was made using the formula for estimating the proportion for a finite population. In this case, the population was the number of PHC professionals with bachelor’s degree working in MG (*n* = 24,026) [13], the sample error was 5% and the confidence level was 95%, resulting in a target sample of 379 professionals.

### Data collection

Data collection was performed using an electronic questionnaire via SurveyMonkey®. The average time to respond the questionnaire was 16 min. The questionnaire was developed by the researchers following guidelines [14, 15] and relevant research on the subject [3, 10, 16, 17]. This research considered the conceptual and operational definition of the frailty phenotype proposed by the Cardiovascular Health Study (CHS) [17]. Frailty is a decrease in the body’s homeostasis and resistance to stressors, resulting in a cumulative decline in multiple systems, causing vulnerability to adverse clinical outcomes [17]. The phenotype is composed of the following criteria: unintentional weight loss, self-perceived fatigue, low level of physical activity, reduced muscle strength, and slow gait speed. An individual is considered frail when presents three or more criteria, pre-frail with one or two criteria, and non-frail/ robust when none of the criteria are present [17].

The questionnaire consisted of Part I, characterization of the professional using sociodemographic and work-related information; and Part II, knowledge regarding the frailty syndrome. Part II had an open question, affirmative sentences with a five-point Likert scale, multiple choice questions and checkboxes questions. Some questions had randomized responses to avoid order bias and marking by visual indication. Regarding the configuration, it was not possible to leave questions blank or return to the previous page, and the professional was warned with an error alert when this occurred.

The dependent variable self-reported knowledge was created from the first question in the questionnaire, in which the professional was asked “Which of the options below best describes your knowledge on the frailty syndrome in older adults?” The categories were reclassified into very little/ no knowledge (vaguely and never heard about it) and some knowledge (some, good and very good). The dependent variable theoretical knowledge was represented by the open question “Describe what you understand to be the frailty syndrome in older adults”. Content analysis was performed by two independent researchers and consensus for disagreements with a third one. After analyzing the content of the responses based on the conceptual and operational definition of the frailty phenotype [17], this variable was classified as correct or incorrect/don’t know. The dependent variable of practical knowledge was analyzed by the response to a clinical case. We developed three clinical cases representing each profile of the frailty phenotype based on the presence or absence of the five criteria [17]. First, the acronyms of three older adults in each clinical case were randomly presented. After choosing one of the acronyms, the information for that case was made available to the professional for classify the older adult as “frail,” “pre-frail,” “robust” or “I don’t know,” and then the variable was reclassified as correct or incorrect/don’t know.

Other content covered regarding frailty were: myths, general knowledge of assessment tools to identify frailty (no/ yes- which?), frailty phenotype, care practices in PHC; use of the *Caderneta de Saúde da Pessoa Idosa* [Older Adult Health Handbook] distributed by the Brazilian Ministry of Health [18], evidence on treatment, strategies to improve care, barriers that can hinder the implementation of actions and ways of finding information.

To check the questionnaire’s feasibility its first draft was sent to ten professionals with experience in geriatrics/gerontology or public health. The report was discussed, and changes were made for the pilot phase. The pilot aimed to verify the applicability in the target population with a convenience sample of 33 PHC professionals from a single municipality in MG. The questionnaire showed good evaluation and general understanding by the professionals.

Data collection was performed electronically for a period of one month, from September 23 to October 23, 2019. The Council of Health Secretaries of the state of Minas Gerais (COSEMS/MG) sent an email to municipal managers containing the invitation to participate in the study, together with a link to access the questionnaire, who then forwarded it to PHC professionals.

In order to guarantee the adhesion and representativeness of professionals throughout the MG State, all PHC professionals had the same chance of receiving the invitation, and weekly monitoring of the number of respondents per municipality was conducted. The study had the participation of municipalities from all five geographic regions of MG, with good distribution (around 20–30% of municipalities per region).

### Statistical analysis

SurveyMonkey® data was extracted and analyzed in SPSS 25.0 with a 5% significance level for statistical tests and 95% confidence interval. Descriptive analysis of the data was performed.

The agreement between dependent variables (self-reported, theoretical and practical knowledge) was performed using the kappa coefficient (2 × 2 table). The strength of agreement was determined to be from > 0.9, almost perfect, to 0.0, absent [19].

Inferential statistics were applied to test the set of variables (sociodemographic and work-related data from professionals) related to the lack of knowledge on the syndrome. For association analyzes, the Chi Square, Fischer or Student t tests were applied. The independent variables with association (*p* <0.05) with some type of knowledge were tested in a univariate logistic regression model and those with significant values (*p* <0.05) were included in a multivariate logistic regression model by the stepwise method.

## Results

Eight hundred fifty-three individuals accessed the research link, 244 were ineligible, 79 were excluded for not signing the consent form and 45 for not accessing the second part of the questionnaire related to frailty knowledge. The final sample consisted of 485 professionals, the majority were nurses (62.9%), female (83.3%), with a mean age of 35.9 ± 7.2 years old, who had been working for 6.76 ± 5.70 years in PHC and caring for 11 to 50 older adults (62.7%) per week (Table 1). Between those included forty-three professionals did not answer all the questions from the Part II of the questionnaire. There was no difference on sociodemographic and work-related data between those included and the ones that answered only the first part of the questionnaire.

**Table 1:** Sociodemographic and work-related data of primary health care professionals (*n* = 485)

Variable	n (%)	Mean ± SD
**Sex**		
Female	404 (83.3)	
**Age range**		
20-29 years	78 (16.1)	
30-39 years	282 (58.1)	
40-49 years	100 (20.6)	
≥50 years	25 (5.2)	
**Profession**		
Nurse	305 (62.9)	
Physician	45 (9.3)	
Physical Therapist	37 (7.6)	
Physical Educator	25 (5.2)	
Psychologist	19 (3.9)	
Dietitians	19 (3.9)	
Dentistry	18 (3.7)	
Social worker	11 (2.3)	
Pharmacist	3 (0.6)	
Ocuppational Therapist	3 (0.6)	
**Years of working**		9.9 ± 6.3
0-4 years	86 (17.7)	
5-9 years	162 (33.4)	
10-14 years	156 (32.2)	
≥15 years	81 (16.7)	
**Academic degree**		
Bachelor’s degree	154 (31.8)	
Specialization	314 (64.7)	
Master’s Degree	16 (3.3)	
PhD	1 (0.2)	
**Specialization in PHC**		
Yes	201 (41.4)	
**Specialization in older adult care**		
Yes	15 (3.1)	
**Training/Course in older care in PHC**		
Yes	154 (31.8)	
**Years of working at PHC**		6.7 ± 5.7
0-4 years	207 (42.7)	
5-9 years	146 (30.1)	
10-14 years	87 (17.9)	
≥15 years	45 (9.3)	
**Number of older adults cared for per week**		
1-10	87 (17.9)	
11-50	304 (62.7)	
51-100	63 (13.0)	
≥101	31 (6.4)	

*PHC* Primary Health Care

**Fig. 1:**
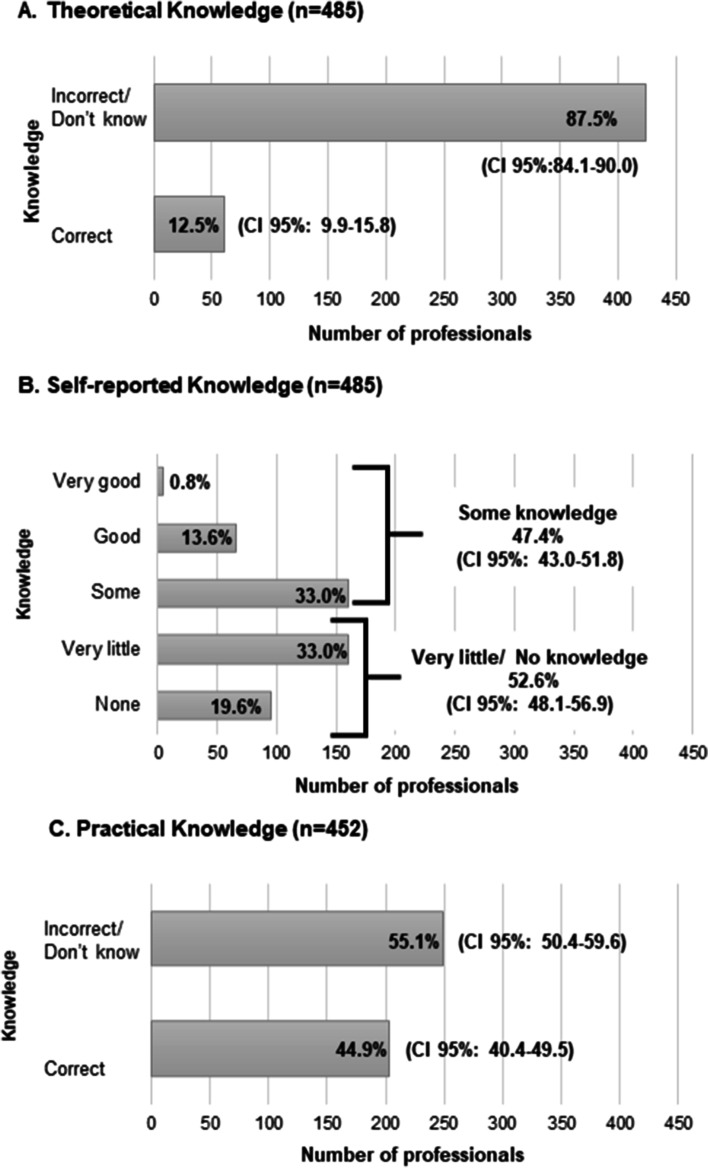
Theorical (**A**), self-reported (**B**) and practical (**C**) knowledge on the frailty syndrome among health professionals

In general, the sample had little knowledge concerning frailty (Fig. 1). Theoretical knowledge showed the worse result, with 87.5% (*n* = 424) of the professionals describing the syndrome incorrectly. When presented the conceptual definition of frailty, the vast majority of the professionals (*n* = 440; 91.9%) strongly agree (56.6%) or agree (35.3%) with this concept, 4.1% (*n* = 20) neither agree or disagree, and 4% (*n* = 19) disagree (2.7%) or strongly disagreed (1.3%). Regarding practical knowledge most professionals (*n* = 249; 55.1%) could not identify the clinical case. The clinical case describing a pre-frail older adult presented a greater number of correct responses (*n* = 95 of 165; 57.6%), while the frail (*n* = 56 of 147; 38.1%) and robust cases (*n* = 52 of 147; 37.1%) showed similar percentages for correct responses. Nearly half the professionals self-reported (*n* = 255; 52.6%) very little/no knowledge.

Among six myths concerning frailty, the professionals erroneously agreed with half the statements and correctly disagreed with the other half. The myth that showed the largest number of erroneously agreements was “frailty is a natural part of the aging process” (*n* = 399; 83.3%), while that with the largest correct disagreements was “provision of care for frail older adults should only be performed by specialists” (*n* = 344; 72.9%) (Table 2).

**Table 2:** Myths and practical approaches concerning the frailty syndrome among primary health care professionals

	Strongly disagree	Disagree	Neither agree or disagree	Agree	Strongly agree
**Misconceptions regarding fraity**					
Frailty is a natural part of the aging process. (*n*= 479)	22 (4.6%)	42 (8.8%)	16 (3.3%)	237 (49.5%)	162 (33.8%)
Frailty is synonymous of disability, comorbidity and advanced age. (*n*= 449)	107 (22.3%)	89 (18.6%)	38 (7.9%)	149 (37.4%)	66 (13.8%)
Every frailty assessment is complex, requires specific knowledge and takes a lot of time to apply. (*n*= 479)	41 (8.6%)	82 (17.1%)	51 (10.6%)	172 (35.9%)	133 (27.8%)
Once the frailty syndrome is installed in older adults, its reversal is not possible.(*n*= 479)	139 (29.0%)	147 (30.7%)	70 (14.6%)	106 (22.1%)	17 (3.5%)
I believe that the evaluation of the frailty syndrome should be performed only by specialists in the care of older adults.(*n*= 472)	178 (37.7%)	120 (25.4%)	39 (8.3%)	99 (21.0%)	36 (7.6%)
I believe that the provision of care for frail older adults should be performed only by specialists in the care of older adults. (*n*= 472)	222 (47.0%)	122 (25.9%)	34 (7.2%)	67 (14.2%)	27 (5.7%)
**Practices regarding frailty in Primary Care**					
I feel prepared to identify frail older adults who seek care at the primary health care where I work. (*n*= 472)	35 (7.4%)	97 (20.7%)	43 (9.1%)	237 (50.2%)	60 (12.6%)
In the primary health care where I work, instruments are used to detect and screen for frailty in older adults.(*n*= 472)	142 (30.1%)	101 (21.4%)	71 (15.0%)	124 (26.3%)	34 (7.2%)
I feel prepared to provide care to frail older adults at the primary health care where I work. (*n*= 472)	44 (9.3%)	104 (22.0%)	50 (10.6%)	207 (43.9%)	67 (14.2%)
At the primary health care where I work, activities aimed at preventing frailty in older adults are offered.(*n*= 472)	97 (20.6%)	80 (16.9%)	43 (9.1%)	171 (36.2%)	81 (17.2%)
I believe that every older adult should be periodically evaluated for the frailty syndrome in the primary health care service.(*n*= 479)	4 (0.8%)	7 (1.5%)	15 (3.1%)	95 (19.8%)	358 (74.8%)
I believe that recognizing the state of pre-frailty is crucial in providing care to older adults in primary health care.(*n*= 472)	7 (1.5%)	16 (3.4%)	20 (4.2%)	100 (21.2%)	329 (69.7%)

Concerning daily practice on PHC, most of the professionals feel prepared or strongly prepared to identify (*n* = 297; 62.8%) and to provide proper care (*n* = 274; 58.1%) for frail older adults. Also, the majority of the professionals disagree or strongly disagree that instruments are used to screen for frailty (*n* = 243; 51.5%) but agree or strongly agree that activities aimed at preventing syndrome are provided (*n* = 252; 53.4%) on PHC (Table 2).

Most of the professionals (*n* = 363; 77.9%) had no knowledge regarding frailty instruments. Among those who responded that they knew (*n* = 103), only 29.1% (*n* = 30) correctly cited a frailty assessment, the remainder cited nonspecific (*n* = 33; 32.0%), physical functional (*n* = 25; 24.3%) and mental health assessments (*n* = 15; 14.6%). The frailty instruments cited were the Clinical Functional Vulnerability Index-20 (IVCF-20) (*n* = 9), the Edmonton Frail Scale (EFS) (*n* = 6), the frailty phenotype (*n* = 5), calf circumference (i = 5), the Vulnerable Elders Survey-13 (VES-13) (*n* = 3) and an isolated phenotype criterion (*n* = 2).

The majority of the professionals (*n* = 343; 73.6%) reported not using the government’s handbook on older adult health. Of the 103 professionals who reported using it, only 24 (19.5%) were able to identify questions/ tests contained in the handbook that could assist in screening for frailty. The VES-13 (*n* = 15), calf circumference (*n* = 5) and weight loss (*n* = 4) were mentioned.

**Fig. 2:**
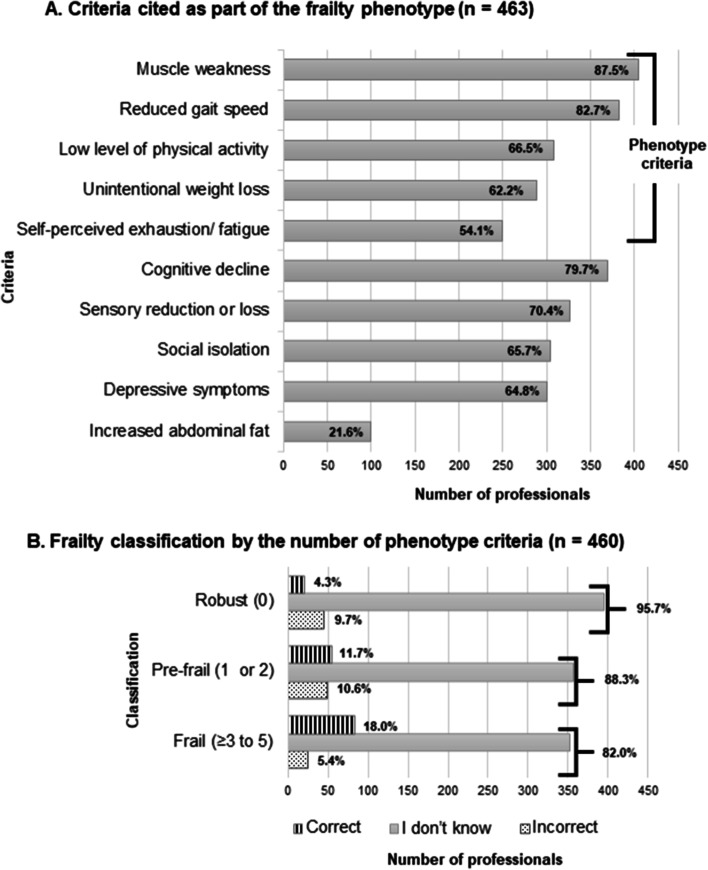
Knowledge on the frailty phenotype criteria (**A**) and classification (**B**) among health professionals

Among a list of 10 criteria, only 31.8% (*n* = 147) identified the set of five criteria of the frailty phenotype proposed by the CHS. The most cited criterion was muscle weakness (*n* = 405; 87.5%) and the least was self-perceived fatigue (*n* = 250; 54.1%). The item most mentioned, which does not form part of the phenotype, was cognitive decline (*n* = 369; 79.7%) (Fig. 2 A). The vast majority of the professionals reported not knowing the number of criteria necessary for frailty classification (Fig. 2B).

Among 16 possible actions for prevention and intervention in frailty, the professionals were asked to identify five scientific records of evidence. The most frequently cited were family involvement (*n* = 312; 70.6%). Interventions with a high level of evidence, such as strength exercises (*n* = 165; 37.3%) and nutritional supplementation (*n* = 69; 15.6%), were rarely mentioned (Additional file 1A).

Among 10 strategies to improve care regarding frailty in PHC, the professionals were asked to choose the three most important. The most frequently cited were continuing education for health professionals (*n* = 314; 71.0%), the implementation of screening for frailty (*n* = 209; 47.3%), and training for community health workers (*n* = 177; 40.0%) (Additional file 1B). Among 13 barriers for the implementation of actions in PHC related to frailty, the most frequently cited by professionals were the lack of skilled professionals (*n* = 279; 63.1%), the lack of knowledge concerning frailty (*n* = 253; 57.2%) and the lack of family and community involvement (*n* = 191; 43.2%) (Additional file 1C).

The vast majority of professionals (*n* = 439; 99.3%) are interested in receiving information on the syndrome. The professionals were asked to indicate three ways of acquiring such knowledge from a list of 11 items. The most frequently cited were a face-to-face (on-site) training course (*n* = 278; 62.9%), an online training course (*n* = 194; 43.9%), and handbooks/instructional material (*n* = 186; 42.1%) (Additional file 2).

No statistical agreement was observed between practical knowledge and theoretical (*k* = 0.036; *p* = 0.275) and self-reported knowledge (*k*=-0.064; *p* = 0.170). There was an absence of agreement between self-reported and theoretical knowledge (*k*=-0.112; *p* < 0.001).

No association was observed between theoretical knowledge and sociodemographic and work-related data of the professionals. Practical knowledge was associated with the age group, such that older professionals presented better analysis of the clinical case compared with their younger colleagues (*p* = 0.012), but this did not remain in the univariate model (Additional file 3). Professionals who had some formal education in older adult health (specialization and training courses) showed 6.1 and 2.7-fold greater chance of self-reporting knowledge on the syndrome, respectively, than those who had no such training (Table 3).

**Table 3:** Logistic regression with variables associated with self-reported knowledge of frailty among health professionals

			B	S.E.	Wald	*p*-value	Odds Ratio	CI 95%
**UNIVARIATE**	**Specialization in older adult care**	**No**	Reference					
		**Yes**	2.02	0.76	7.00	**0.008**	1.69	1.69 – 33.95
	**Training/ Course in older care in PHC**	**No**	Reference					
		**Yes**	1.05	0.20	26.86	**>0.001**	2.86	1.92 – 4.26
	**Number of older adults cared for per week**	**1-10**	Reference					
		**11-50**	-0.31	0.42	0.54	0.460	0.72	0.31 – 1.68
		**51-100**	0.35	0.38	0.85	0.357	1.42	0.67 – 3.00
		**≥101**	0,42	0.44	0.90	0.342	1.52	0.63 – 3.62
**MULTIVARIATE**	**Specialization in older adult care**	**No**	Reference					
		**Yes**	1.82	0.77	5.50	**0.019**	6.19	1.35 – 28.40
	**Training/ Course in older care in PHC**	**No**	Reference					
		**Yes**	1.01	0.20	24.40	**>0.001**	2.75	1.84 – 4.10

*PHC* Primary Health Care, *B *regression coefficient, *S.E *standard error, *CI *Confidence Interval

## Discussion

This study revealed that PHC professionals have little knowledge concerning frailty, particularly theoretical knowledge. The lack of knowledge, skills and abilities of health professionals and their relation to promoting adequate care for the older adult population is of concern to the World Health Organization [20]. European Union health policy managers have indicated that there is currently no adequate management of frailty in health services, and this can be attributed in large part to the lack of knowledge of health professionals on this subject [21]. Thus, the lack of knowledge that we observed among the professionals in our study can interfere in actions to identify, intervene and monitor older adults in situations of frailty in the PHC.

Regarding the frailty phenotype, most professionals could not define it, nor its operationalization. In a systematic review [22] of 86 studies, the majority used the one-dimensional conceptual definition of physical frailty (41%) and the operational phenotype (71%) proposed by the CHS, which was the same used by our research. Another systematic review found that the frailty phenotype was the most highly cited assessment, and used specially to evaluate risk of adverse outcomes, and the etiology and the biomarkers of frailty [23]. However, this strictly biomedical model was extended to a comprehensive and dynamic model that has a “biopsychosocial” basis, in which frailty is influenced not only by the physical component, but also by social, cognitive, economic and behavioral factors [24], and can be measured by the Deficit Accumulation Index.

The professionals agree with some myths and stereotypes related to frailty. Even among multidisciplinary geriatric and gerontologic health professionals, there is confusion about the term frailty, with more than 50% identifying “frailty” related to levels of functional independence and to the presence of medical conditions [9]. In the Brazilian Longitudinal Study of Aging (ELSI-Brazil) [25], involving 8,556 individuals, 30% of the participants presented concomitant comorbidity, disability, and frailty, while 26% presented frailty alone. Thus, it is important that professionals can distinguish these three concepts and understand that although they can coexist, frailty has a specific and independent biological foundation [8].

Despite the availability of numerous instruments for assessing frailty, the professionals in our study mostly reported having no knowledge of these. In a systematic review [26], 51 instruments were found to assess frailty, nine of which have already been translated into and validated for Brazilian Portuguese. Even though it was the most cited instrument, the IVCF-20 was mentioned by very few of the professionals (*n* = 9). This is especially concerning considering that the questionnaire is disseminated in trainings given by the Ministry of Health [27]. Using the Comprehensive Geriatric Assessment as the gold standard, the IVCF-20 showed sensitivity of 90.0% and specificity of 70.0% in screening for frailty in the Brazilian population [28]. A systematic review [29] on screening instruments for frailty in low- and middle-income countries reported that among 27 studies conducted in Brazil, the majority (63%) used the CHS phenotype. Still, there is no consensus of which is the best method to screen for frailty. Thus, it is important to establish the use of the same frail assessment to facilitate and standardize the work in PHC.

Among the frailty phenotype criteria, the least mentioned by the professionals was self-perceived fatigue. The ELSI-Brazil [25] demonstrated that self-perceived fatigue was the most common criterion (28%) among frail older adults. Since fatigue is a highly prevalent criterion in frail older adults, it deserves greater attention in clinical practice.

In this research, we observed low adherence by professionals in the use of the government’s handbook on older adult health. In addition, few professionals identified items in the handbook that could assist in screening for frail, such as weight loss, body mass index and calf circumference. Lack of use of the handbook was also observed in a qualitative study involving PHC managers and professionals, which can be explained by the lack of training and difficulties in filling it out [30]. Use of the handbook by PHC professionals enables the monitoring of older adult health, while for health managers, it provides statistical data on the older adult population that enable the organization of actions, the establishment of goals and monitoring the results [30].

Concerning practical knowledge, the majority of the professionals did not know how to classify the older adult in the clinical case with regard to frailty and only partially agreed that they are prepared to identify and to provide care for frail older adults in PHC. Sixty-two geriatricians were presented clinical cases for recognize frailty, in which frail individuals were identified as those with alterations resulting from diseases in addition to the changes inherent to aging, while individual illnesses, or the isolated presence of disability, were not considered sufficient to identify frail older adults [8]. In a qualitative study [31], health professionals described using their own methods to identify frail older adults, since they often do not use standardized assessments because they do not know they exist, or do not know how to relate them to practical situations.

Although there is growing evidence for frailty treatment [10, 16], the professionals in our study showed minimal knowledge of it. A systematic review [16] recommended, with a high level of evidence (GRADE A), physical exercise programs performed in groups and nutritional supplementation for the prevention of pre-frailty and the progression of frailty in community older adults. Another systematic review [10] involving 15,690 older adults revealed that muscle strength training and protein supplementation are interventions for frailty that show greater relative effectiveness and ease of implementation in the context of PHC. However, it is not clear whether these interventions improve frailty itself or ameliorates only a specific frailty criterion. Not recognizing this evidence means professionals fail to provide the best care for older adults, which in the long run generates greater costs for the system and more illness in this population.

The professionals highlighted family involvement as the main evidence in the frailty care, and at the same time, one of the main causes of difficulties in the implementation of actions in the PHC practice. There are no data regarding the benefits of family engagement in caring for frail older adults [32]. However, for care centered on the frail older adult to be effective, formal and joint engagement of all those involved in the process is necessary, i.e., the older adult, their support network (family members, caregivers, neighbors) and the health team [32].

The professionals indicated both the lack of skilled professionals and the lack of knowledge on the subject as the main difficulties in implementing actions for frailty in the PHC. Also, they indicated professional training as the main strategy for improving care for frail older adults. Health professionals reported that there was no shared understanding between the areas of expertise regarding frailty [33]. There is an urgent need for interdisciplinary gerontological education of professionals in response to population aging. Professors of courses in the health field have indicated the need for the inclusion of content on geriatrics/gerontology in the curricula at both undergraduate and graduate levels [34]. Moreover, even the courses that offered this content presented limitations, including: a reduced number of hours for practical training, a shortage of educators trained in geriatrics/gerontology, and stereotypes concerning the older adult population (e.g. lack of exposure to individuals without disabilities or not sick) [34].

In Brazil from the end of the 90 s onwards, there was an increase in geriatric/ gerontology training with policies to include aging content in the undergraduate curriculum in all health disciplines [35]. In the field of aging, we have undergraduate Gerontology courses, specialization courses in all Brazilian states [35], and free short courses and continuing education available by partners of the Ministry of Healthy. However, in our study, although participation in specialization or in courses of older adult care were the only factors associated with greater self-reported knowledge of the syndrome, there was no evidence that this conferred greater theoretical and practical knowledge to the professionals, which raises question regarding the quality and applicability of the contents of the courses on offer. Representatives of health councils in São Paulo, Brazil, indicated that public service health professionals present a fragmented view of older adults, thus preventing comprehensive care for this population [36]. The authors highlight that to change this scenario, it is important that any training is practical and that monitoring the implementation of knowledge acquired in the daily life of health services is constant [36].

Our study indicates that the professionals showed interest in receiving information about frailty, especially in a face-to-face format. Community care staff reported that practical and multidisciplinary training on frailty that simulates the reality of the work environment, instead of a purely theoretical session, facilitates the learning process and encourages discussions between the specialties [33]. The second option was online training. Online training courses facilitate democratic access to knowledge by meeting individual needs, both in relation to the pace of learning and the time available to the professional [37]. Corroborating with our results, a qualitative study with 31 PHC professionals found that the preferred educational training for frailty were online or face-to-face with opportunities to learn practical skills [38]. However, a systematic review [39] demonstrated that there is a lack of evidence regarding the effectiveness of educational programs on frailty for health professionals. A single-day training course about frailty for PHC professionals showed improvements in attitudes, knowledge, and everyday practices upon completion and 3 months after [40].

Self-reported, theoretical and practical knowledge proved to be distinct constructs. The professional may have theoretical knowledge, while not having the practical experience to implement it in care situations. Likewise, a lack of theoretical knowledge can lead to actions based on the professionals’ practical experiences, which do not always reflect the best current evidence. Since self-reported knowledge is influenced by subjective factors, we may have situations in which the professional has a lot of theoretical or practical knowledge, but their self-perception is low or the opposite, where confidence substitutes good practice. To increase each of these forms of knowledge, different educational strategies are needed.

Obtaining data by electronic questionnaire is a limitation of this study, since it is not possible to ensure that professionals did not consult research sources on the subject during its completion. In addition, even though nursing is the largest professional group in Brazilian PHC, we believe that the low participation of professionals from other areas in our sample is also a limitation, in view of the importance of the multidisciplinary team for the best care for the syndrome. The fact that the study sample is relatively young (mean age 35.9 years old) might be also considered as a limitation. In this sense, younger professionals are more likely to have received more information during their education about the syndrome, as the definition of frailty was conceived in earlies 2001 [17]. Considering those eligible, our response rate was 79.6%. Based in the literature of low response rate for electronic questionnaires from 17% [41] to 35% [42], and the fact that professionals may not be comfortable to give work-related information, we believe the responsiveness rate is a strong point of our research.

## Conclusions

This study revealed that health professionals from primary care have little knowledge on the frailty syndrome, whether self-reported, theoretical or practical. These three types of knowledge proved to be distinct constructs. Even though professionals with a degree or training in the field of older adult health self-reported greater knowledge compared with the remainder of our sample, this difference did not reflect in greater theoretical and practical knowledge. It is important that PHC professionals develop skills to identify the particularities and needs of frail older adults, anticipating future demands and the risks of worsening health in this population.

## Supplementary Information

**Additional file 1.** Care actions for frailty in primary health care. (A) Evidence (B) Strategies (C) Barriers.

**Additional file 2.** Formats for receiving information on the frailty syndrome by health professionals.

**Additional file 3.** Associations and univariate analysis of sociodemographic and work-related variables with types of knowledge about frailty among health professionals.

## Data Availability

The data underlying this article are stored in Harvard Dataverse at 10.7910/DVN/K0EKDA. The datasets used and/or analyzed during the current study are available from the corresponding author on reasonable request.

## Abbreviations

EFS: Edmonton Frail Scale
ELSI-Brazil: Brazilian Longitudinal Study of Aging (**E**studo **L**ongitudinal de **S**aúde dos **I**dosos Brasileiros)
IVCF-20: Clinical Functional Vulnerability Index-20 (**Í**ndice de **V**ulnerabilidade **C**línico-**F**uncional-20)
MG: Minas Gerais
PHC: Primary health care
VES-13: Vulnerable Elders Survey-13

## Declarations

All methods were performed in accordance with the relevant guidelines and regulations.
