# Supplementary material for: What do Brazilian health professionals know about the frailty syndrome? A cross-sectional study
Source: BMC Geriatr. 2022 Mar 21;22:232. doi: 10.1186/s12877-022-02927-6 (PMC8939059; doi:10.1186/s12877-022-02927-6)

Additional file 1 Care actions for frailty syndrome in primary care.
(A) Evidence (B) Strategies (C) Barriers

A. Knowledge of evidence of prevention and intervention in the frailty syndrome (n= 442)

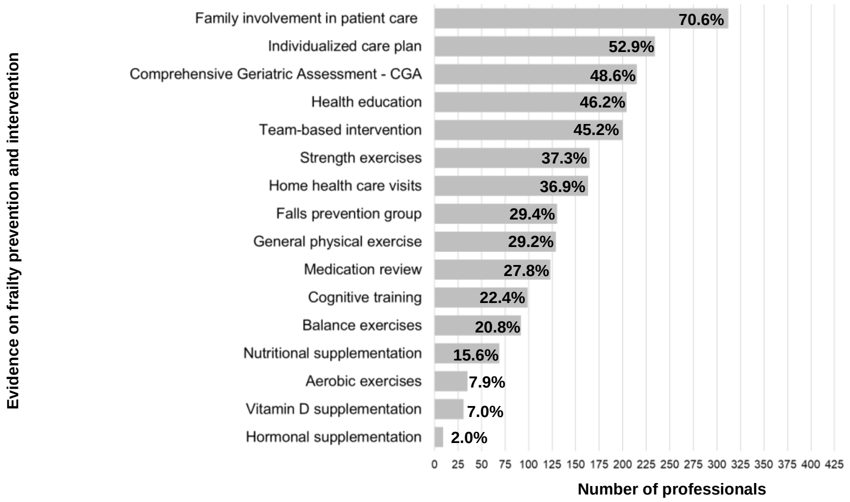

B. Strategies to improve care for frailty syndrome (n= 442)

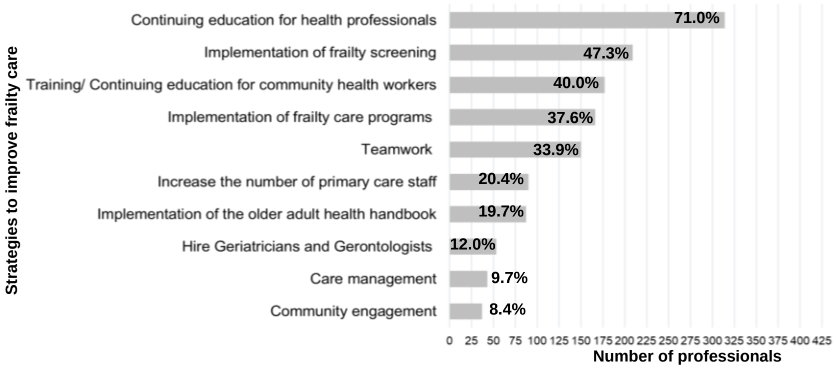

C. Factors identified as barriers to the implementation of actions for frailty (n= 442)

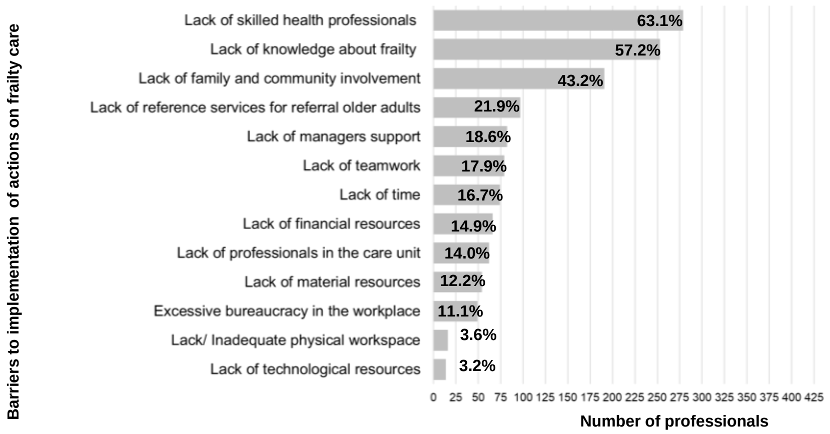

Supplement: Supplementary file 1 — Additional file 1. Care actions for frailty in primary health care. (A) Evidence (B) Strategies (C) Barriers. [file 12877_2022_2927_MOESM1_ESM.pdf]
