# Supplementary material for: What do Brazilian health professionals know about the frailty syndrome? A cross-sectional study
Source: BMC Geriatr. 2022 Mar 21;22:232. doi: 10.1186/s12877-022-02927-6 (PMC8939059; doi:10.1186/s12877-022-02927-6)

Additional file 2- Formats for receiving information on the frailty syndrome by health professionals (n= 442).

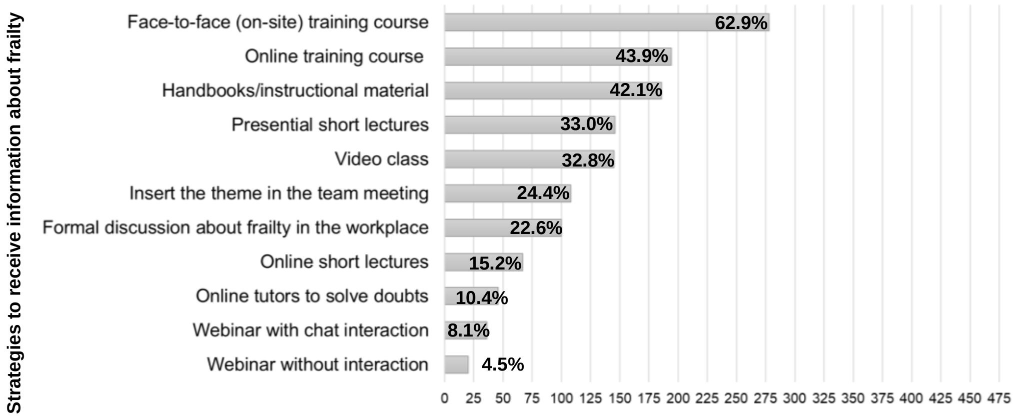

Supplement: Supplementary file 2 — Additional file 2. Formats for receiving information on the frailty syndrome by health professionals. [file 12877_2022_2927_MOESM2_ESM.pdf]
