# Supplementary material for: What do Brazilian health professionals know about the frailty syndrome? A cross-sectional study
Source: BMC Geriatr. 2022 Mar 21;22:232. doi: 10.1186/s12877-022-02927-6 (PMC8939059; doi:10.1186/s12877-022-02927-6)
Supplement: Supplementary file 3 — Additional file 3. Associations and univariate analysis of sociodemographic and work-related variables with types of knowledge about frailty among health professionals. [file 12877_2022_2927_MOESM3_ESM.pdf]

Additional file 3- Analysis between sociodemographic and work-related data with types of knowledge (A) Associations (B) Univariate logistic regression

(A) Associations between sociodemographic and work-related data with types of knowledge

Variable	Self-reported (n=485)		Theoretical (n=485)	Practical (n=452)	
**Sex***	0.731		0.766	0.336	
**Age****	0.095		0.547	0.073	
**Age range***	0.100		0.377	**0.012**	
				**Correct**	**Incorrect/ DK**
**20-29 yrs**				33	41
**30-39 yrs**				106	161
**40-49 yrs**				53	36
**≥50 yrs**				11	11
**Profession***	0.946		0.189	0.254	
**Years of working (range)***	0.387		0.702	0.924	
**Years of working (yrs)****	0.673		0.160	0.945	
**Years of working at PHC (range)***	0.781		0.072	0.433	
**Years of working at PHC (yrs)****	0.373		0.840	0.421	
**Academic degree***	0.078		0.687	0.288	
**Specialization in PHC ***	0.109		0.633	0.845	
**Specialization in in older care ***	**0.002**		0.095	0.139	
	**Very little/ no knowledge**	**Some knowledge**			
**Yes**	2	13			
**No**	253	217			
**Training/Course in older care in PHC***	**0.000**		0.178	0.173	
	**Very little/ no knowledge**	**Some knowledge**			
**Yes**	54	100			
**No**	201	130			
**Number of older adults cared for per week***	**0.044**		0.156	0.327	
	**Very little/ no knowledge**	**Some knowledge**			
**1-10**	57	30			
**11-50**	150	154			
**51-100**	30	33			
**≥101**	18	13			

* Chi-squared test/ Fischer test

**T-student test

PHC: Primary Health Care; DK: Don't know

(B) Univariate logistic regression of age range and practical knowledge of frailty

UNIVARIATE		B	S.E.	Wald	*p*-value	Odds Ratio	CI 95%
**Age range**	**20-29 yrs**	Reference					
	**30-39 yrs**	0.20	0.26	0.57	0.449	1.22	0.72 – 2.05
	**40-49 yrs**	-0.60	0.31	3.59	0.058	0.54	0.29 – 1.02
	**≥50 yrs**	-0.21	0.48	0.19	0.655	0.80	0.31 – 2.08

PHC:Primary Health Care; B: regression coefficient; S.E: standard error; CI: Confidence Interval
